# The meaning of work for hospital unit nurses: a scoping review

**DOI:** 10.47626/1679-4435-2023-1116

**Published:** 2024-11-14

**Authors:** Thiago Portela Caroccini, Alexandre Pazetto Balsanelli, Vanessa Neves

**Affiliations:** 1 Escola Paulista de Enfermagem, Universidade Federal de São Paulo, São Paulo, SP, Brazil; 2 Instituto D’Or de Pesquisa e Ensino, São Paulo, SP, Brazil

**Keywords:** work-life balance, work engagement, nursing, nurses, hospitals, equilíbrio trabalho-vida, engajamento no trabalho, enfermagem, enfermeiras e enfermeiros, hospitais

## Abstract

Over the years, work has become a meaningful activity in the lives of professionals, contributing to social inclusion and personal fulfillment and influencing quality of life. This is particularly true for nurses, for whom work has significant meaning. This study aims to explore how nurses’ recognition of the meaning of their work is critical to their job performance. This scoping review was conducted using the Cumulative Index to Nursing and Allied Health Literature, Latin American and Caribbean Health Sciences Literature, Medical Literature Analysis and Retrieval System Online, and American Psychological Association databases. The review included studies in Portuguese, English and Spanish published between 2016 and 2022. The final sample included 10 studies that reported characteristics related to the meaning of work: 56% were qualitative descriptive studies, 22% were cross-sectional, and 22% did not report their design. Most studies were conducted in Brazil (n = 6), followed by China, Japan, and Namibia. Articles were classified and analyzed according to the following categories: sample, description of values or attributes of the meaning of work, and hospital context. The study concludes that nurses perceive meaning in their work through affirmation; exchange of esteem; sharing of information; trust; autonomy; interpersonal relationships; communication; interpersonal relationships with patients; reciprocity; involvement; job satisfaction; opportunities for learning and development; recognition and moral integrity; affective commitment; and stress related to work hours.

## Abstract (pt)

Ao longo dos anos, o trabalho tornou-se uma atividade repleta de significados na vida dos profissionais, favorecendo a inclusão social e a realização pessoal, com impacto na qualidade de vida, sendo, assim, uma laboração carregada de significado, principalmente para enfermeiros. Este estudo visa verificar como o reconhecimento do significado do trabalho do enfermeiro é importante para o desempenho da função. Este trabalho é uma revisão de escopo realizada nas bases Cumulative Index to Nursing and Allied Health Literature, Literatura Latino-Americana e do Caribe em Ciências da Saúde, Medical Literature Analysis and Retrieval System On-line e American Psychological Association. Foram incluídos estudos em português, inglês e espanhol, no período de 2016 a 2022. Contemplaram a amostra final 10 estudos relatando atributos do significado do trabalho: 56% estudos descritivos qualitativos, 22% transversais e 22% não relataram o desenho. Houve predominância de estudos no Brasil (n = 6), seguidos de China, Japão e Namíbia. Os artigos foram classificados e analisados a partir das categorias: amostra, descrição dos valores ou atributos do significado do trabalho e o contexto hospitalar. Conclui-se que enfermeiros reconhecem o significado do trabalho através de afirmação; troca de apreço; compartilhamento de informações; confiança; autonomia; relação interpessoal; comunicação; relação interpessoal com paciente; relação de reciprocidade; engajamento; felicidade no trabalho; oportunidade de aprendizagem e desenvolvimento; reconhecimento e retidão moral; comprometimento afetivo; e estresse pelo tempo de trabalho.

## INTRODUCTION

Over the years, work has become an activity filled with meaning in the lives of workers, contributing to social inclusion and personal fulfillment, and impacting quality of life. This is particularly true for nurses, whose work is especially meaningful.^1^

The work environment in nursing is notoriously stressful due to constant challenges, and nurses often experience the brunt of this psychological strain. They seek happiness in this environment to ensure job satisfaction, find meaning in their work, and remain engaged and productive. As a result, nurses tend to give of themselves to help others, often working double shifts with physically and mentally exhausting tasks that affect their well-being.^2^

The term “meaning of work” is intuitively defined, considering the knowledge gained throughout life, including common sense. For work to have meaning, it must be evaluated through subjective interpretation, intertwined with cultural, social, and even pandemic-related factors. Therefore, it is not easy to define, as it is frequently associated with concepts like motivation, commitment, and satisfaction.^3^

Because of the many changes affecting organizations and the world of work, understanding its current meaning is a significant challenge for managers; however, we believe that this understanding is also necessary in other fields and disciplines. The path of development has led to the creation of practices that require interdisciplinary actions and new work frameworks; therefore, work must be understood as dynamic and social.^4,5^

The nursing work process is not limited to the physical environment, but also includes other multifactorial and interrelated elements such as team communication, perceptions of one’s role and function, organizational and social support, workload and pace, power dynamics, autonomy, commitment, and the availability and quality of resources and processes for providing care. These aspects have a significant impact on the meaning of work.^5-7^

Managers and nurses, as team leaders, must be able to identify the presence or absence of characteristics that support nursing professionals, allowing them to implement actions that improve the work environment, thereby enhancing the meaning of work and, consequently, improving institutional outcomes.^2^

This discussion highlights the importance of understanding the meaning of work for nurses and addresses a gap in the literature regarding the global context and evaluation of the meaning of work for nurses. This study aimed to understand how nurses perceive the meaning of their work within their professional environment.

## METHODS

### SCOPING REVIEW

The study was conducted according to the Preferred Reporting Items for Systematic Reviews and Meta-Analyses (PRISMA) checklist, which guided the organization of the information. The present review included: formulating the review question, searching the literature for primary studies, extracting data, assessing the primary studies, analyzing and synthesizing the results, and presenting the review. As a starting point, the review question was formulated as follows: “How do nurses perceive the meaning of their work in their professional environment?” The PCC (P: population, C: concept, C: context) methodology was used to develop this question, with the population defined as nurses, the concept as meaning of work, and the context as the hospital environment.

Searches for primary studies were conducted in the following databases: Cumulative Index to Nursing and Allied Health Literature (CINAHL), Latin American and Caribbean Health Sciences Literature (LILACS), and Medical Literature Analysis and Retrieval System Online (MEDLINE) through the National Library of Medicine National Institutes of Health (PubMed) and the American Psychological Association (PsycNET).

For the search, the review authors selected controlled and uncontrolled descriptors from the Medical Subject Headings (MeSH) and Health Sciences Descriptors (DeCS) terminology, including “equilíbrio trabalho-vida” (“work-life balance”); “engajamento no trabalho” (“work engagement”); “enfermagem” (“nursing”); “enfermeiras e enfermeiros” (“nurses”); and “hospitais” (“hospitals”). They also searched for the terms “sentidos do trabalho” e “significado do trabalho” (“meaning of work”).

To ensure a comprehensive search, the controlled descriptors were used in various ways, both individually and in combination with each other, using the boolean operators AND and OR. The search in the electronic databases included articles focused on investigating the meaning and significance of work among nurses working in hospital settings, published between 2016 and 2022, in Portuguese, English, and Spanish.

The exclusion criteria consisted of studies that did not investigate the meaning and significance of work among nurses. Articles identified as reflective/theoretical pieces, editorials or letters to the editor, as well as commentaries, theses, and dissertations were also excluded.

As the topic focuses specifically on the meaning of work, some studies addressed the topic as “meaning of work”; therefore, the search was conducted using both terms, especially in English. Duplicate articles were excluded from the sample. Search for primary studies on the databases was conducted in July 2022 by one of the review authors. Study selection was performed independently by two review authors, with an agreement rate of more than 80%. In cases of disagreement, a third review author made the final selection. All selections were made by using Rayyan software.

Data extraction was performed independently by using a data collection form to organize key information from the studies. This form included details such as publication identification (article title, language, authors, publication date), journal of publication, and methodological characteristics (type of publication, type of research, sample/participants, and outcomes).^8^

For the analysis of the material, the information was grouped and categorized according to author, year of publication, country, interventions, type of research, number of participants, and results related to the meaning of work. The results were analyzed descriptively. Comparisons were then made to highlight differences and similarities between the studies.

## RESULTS

**Figure 1:**
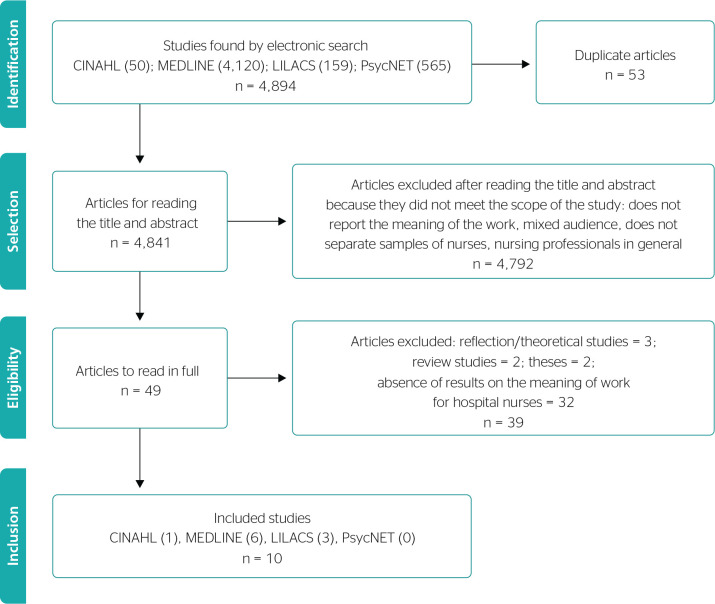
Flowchart of the study selection process, São Paulo, SP, Brazil, 2022. CINAHL = Cumulative Index to Nursing and Allied Health Literature; LILACS = Literatura Latino-Americana e do Caribe em Ciências da Saúde; MEDLINE = Medical Literature Analysis and Retrieval System on-line; PsycNET = American Psychological Association.

The PRISMA flowchart illustrates the selected articles and the sequence followed to include the 10 articles deemed relevant for analysis according to the proposed criteria (Figure 1).

Table 1 provides a description of the characteristics of the articles included in the scoping review.

**Table 1:** Characteristics of the studies included in the scoping review, São Paulo, SP, Brazil, 2022

Author/country	Interventions	Design and sample	Results
Rodrigues et al.^3^ Brazil	Printed questionnaire applied in two stages with a 15-day interval to avoid multicollinearity. Structured instrument measuring Affective Commitment, Stress from Anxiety, Stress from Time, and Meaningfulness Work.	Study type not reported 51 nurses	Variables: meaningfulness of work; autonomy; learning and development opportunities; recognition and moral rectitude; affective commitment; and time stress.
Norikoshi et al.^6^ Japan	Personal interviews with a semi-structured questionnaire lasting 30-40 minutes.	Qualitative study 32 nurses	Attributes that influenced: affirmation; exchange of appreciation; unrestricted sharing of information; ability to trust; access to force; and altruistic reciprocity.
Sobroza et al.^9^ Brazil	Personal interviews with a semi-structured questionnaire, no set time limit.	Qualitative study 5 nursing professionals, roles not detailed	Description of the Meaning Guides: the uncertainties that surround life; the positive value of the job, with the challenge of caring in order to feel a sense of accomplishment; the feeling of a lack of meaning in people’s lives; the bond with the patient; being able to express joy and sadness; listening and being open to the unpredictable; and feeling neglected through exhaustion from repetitive work associated with inadequate pay.
Salimena et al.^10^ Brazil	Open interview, recorded, mediated by empathy.	Qualitative study 3 nurses	Attributes that influenced: interpersonal relationship; communication; conflicts; interpersonal relationship with patient; and reciprocal relationship.
Costa et al.^11^ Brazil	Personal interviews with a semi-structured questionnaire, no set time limit.	Qualitative study 22 nurses using the snowball technique	Variables: caring for people who may be stigmatized due to their injury; having autonomy; and being an entrepreneur.
Pimenta et al.^5^ Brazil	EIPST and structured questionnaire for work characteristics.	Cross-sectional study 152 nurses	Variables: professional fulfillment and freedom of expression; lack of recognition; unit/sector and work schedule.
Zhu et al.^7^ China	Structured questionnaire conducted in two phases: an online questionnaire on demographics and prosocial motivation before starting the shift, followed by a daily 5-minute online survey for 5 consecutive working days during the week on duty.	Study type not reported 66 nurses	Variability in passion for work has a direct impact on its meaning and performance. The greater the vocation to be a nurse, the better the performance and meaning of their work. In practice, the greater the drive for resuscitation, to save lives, the greater the meaning of their work.
Franco et al.^1^ Brazil	Personal interviews with a semi-structured questionnaire with relevant questions: main motivations for developing work; factors that bring suffering and pleasure; and the meaning of being a nurse in the studied institution.	Qualitative study 50 nurses	The motivation at work is a factor influencing the meaning of work, generated through the provision of quality assistance. The recognition of the nursing team, the multi-professional team and, above all, the patients are the items that give the most meaning to their work.
Ogata et al.^12^ Japan	Questionnaires used: PES-NWI, Followership, Psychological Empowerment (PE) Instrument, CICS29, CVF, and Negative Acts Questionnaire Revised.	2-year longitudinal study 7,849 nurses	The characteristics of the work environment are decisive in improving engagement and meaning, as well as the relationship between teams, the health and fatigue of the professional at work and the number of nurses suitable for care.
Ilmalwa et al.^2^ Namibia	Application of questionnaires: WES; SWLS; SHS; and WLQ.	Cross-sectional study 142 nurses	The results indicate that the meaning of work is not related to life satisfaction, but it is negatively related to work engagement and there is a significant relationship between happiness and the meaning of work.

CICS29 = Chiba Interdisciplinary Competency Scale; CVF = Competing Values of Framework; EIPST = Escala de Indicadores de Prazer e Sofrimento no Trabalho; PES-NWI = Practice Environment Scale of the Nursing Work Index; SHS = Subjective Happiness Scale; SWLS = Satisfaction with Life Scale; WES = Work Engagement Scale; WLQ = Work Limitations Questionnaire.

## DISCUSSION

This study aimed to understand the key factors influencing the meaning of work for hospital nurses. Among the primary contributions to the phenomenon is the understanding of work meaning as a means to enhance the relationship between the organization and the individual. The meaning of work has been utilized by organizations as a foundation for employee engagement and motivation, in pursuit of better institutional outcomes. Improving the concept of the relationship between the organization and the individual in the workplace is essential for understanding the individual demands and values of nurses.^1,3,5^

The meaning of work refers to the representations it assumes, and its value as perceived by the nurse, acting as a mediator in the individual’s relationship with their world. From this perspective, a nurse’s orientation towards their work is tied to their vocational calling, aligned with what they seek for themselves and the intentions that guide their actions.^1,7^

Values are a central concept in the meaning that nurses attribute to their work. Beyond normative beliefs and the role work plays in their lives, which motivate them to work, individual values are crucial for a comprehensive understanding of the concept of work meaning. These values relate to the expectations and rewards obtained through work.^12-15^

Nurses are challenged to perform their duties and fulfill their responsibilities in environments that may differ significantly from ideal conditions. In this context, a vocation for the job plays an important role. To cope with these vulnerabilities, nurses seek rewards beyond financial compensation or job stability. Finding meaning in their work is also essential for managing the inherent challenges of nursing.^12,14,16,17^

Workplace characteristics-whether related to care provision or organizational policies, leadership, and interpersonal relationships-moderate the meaning of work. The relationship with managers, and how they treat, support, and recognize their staff, is crucial for creating a better work environment. This, in turn, significantly influences nurses’ individual perceptions and the enhancement of their activities. Key indicators of this influence include effective communication within the team, recognition, appreciation, negotiation freedom with management, autonomy, and adequate compensation for the role performed.^5,18,19^

Indeed, personal relationships with other team members, support from more experienced leaders, and internal opportunities for job change or professional growth are the most impactful factors, proving to be extremely rewarding and influential in improving the work environment. For nurse anesthetists, leader support was also critical, but other variables such as moral value, safety, social support, authority, and responsibility contributed to a better environment, influencing job satisfaction and work meaning. On the other hand, compensation, promotion, and working conditions were the factors that most negatively impacted the environment. For managers, the most influential factors were personal fulfillment and psychological rewards.^14,17,18^

Care settings strongly influence work meaning, depending on work pace, the high demand for physical effort during patient care, the need for skill in performing complex procedures, work shifts, and the demand for quick decision-making.^3,5,10^

Conversely, in critical care settings, nursing practice is often associated with high levels of distress and dissatisfaction with the work itself. In these environments, professionals perform their duties under intense work conditions, high physical demands during patient care, and the need for skillful execution of procedures, alongside the necessity for quick decision making.^5,7,9,15,20^

When discussing work conditions, physical and mental conditions of the team and the structural conditions of the service must be included. These are aspects that influence the environment and, consequently, the meaning of work. Differences between public and private services are factors that should be evaluated. It has been noted that professionals in the Brazilian Unified Health System (SUS) experience lower stress and greater moral recognition and integrity compared to nurses in private institutions. In this case, the meaning may be linked to a social purpose rather than an emotional connection. Other evaluated items, such as affective commitment, time-related stress, and opportunities for learning and development-all with higher averages in private hospitals-also influence work meaning.^3,10,21^

The role of caregiving performed by nurses expresses the essence and meaning of nursing work. Additionally, the professional’s image is influenced by the commitment they express, positively related to their behavior in providing care and potentially influenced by their position or specialty, salary, and rewards. Supervision and communication were also contributing factors.^11^

For operating room nurses, the attributes influencing work meaning included interpersonal relationships, communication, conflicts, interpersonal relationships with patients, and reciprocity. Familiarity among teams, as well as integration, fosters engagement in tasks with a positive attitude and facilitates various positive behaviors and ideas, contributing to improving patient care quality and giving meaning to the nurse’s work. However, in some situations, closed units with close physical proximity between nurses can lead to conflicts and communication difficulties, negatively impacting the work environment.^6,10^

Appreciation during daily activities, mutual assistance, and positive evaluations of one another establish well-being, and nurses’ positive feelings increase, positively impacting work meaning and encouraging cooperative behavior, which facilitates assertive communication and increases engagement. A learning-focused environment, teamwork, and time management with patients facilitate work meaning. Additionally, financial autonomy provides the freedom of choice and decision-making, thus giving meaning to work.^6,12-14,17^

Feelings surrounding caregiving, such as happiness, have a positive relationship with work meaning, as it is observed that most people feel happier when interacting with others and giving them purpose. On the other hand, other elements in nursing practice can negatively impact their activities, diminishing the meaning of work, such as the presence of a hostile environment, high staff turnover, low salaries, and a lack of supplies for adequate care.^2,12,15,21-23^

Work values are the reason for working. These values motivate and give meaning, being both extrinsic and intrinsic. Intrinsic aspects refer to the characteristics of the work activity, such as varied tasks, responsibilities, opportunities to develop skills and abilities, and professional development opportunities. Extrinsic aspects refer to working conditions, such as hours, job security, personal relationships, and income.^21,24-27^

Managers and leaders play a crucial role in establishing strategies that can improve the environment through the values attributed, giving professional meaning to nurses. In practice, the hierarchical superior is substantial not only for human and material resource management but also for effective communication and interrelation among professionals, especially in conflict mediation. However, some situations are beyond their leadership, such as workload, which detracts from meaning. Other factors, such as the lack of individualized attention, misunderstanding of the needs of each department, working conditions, compensation offered to health care workers, and staff shortages, harm work meaning.^2,6,7,10,14,21^

When a nurse performs meaningful work, they build their identity, value, and dignity, contributing to the improvement of their living conditions. Institutions must offer their professionals the opportunity to perform meaningful work and to develop and practice their vocation and skills, exercising their judgment and autonomy, alongside interpersonal relationships. The organization plays a fundamental role in guiding and modifying behaviors so that employees are led to develop positive attitudes toward their work meaning.^2,6,7,10,19-21,27-31^

Therefore, a better understanding of the multiple dimensions involved in the meaning of work for nurses and the mechanisms that can influence it is important. For work meaning to be positive, managers and institutions need to pay attention to situational cues that send clear signals about the needs of professionals, and use these insights to develop policies that contribute to the promotion of healthy environments, making work meaning a positive aspect for both the professional and the institution, ultimately improving the quality of services provided.^2,6,7,11,22-24,27-33^

This study provided important findings and did not attempt to exhaust the discussion on the topic. Since the health field promotes social interaction through the daily activities of workers, this relationship consequently generates positive or negative situations. Therefore, further studies are recommended to include other nursing professionals and hospitals of different sizes to verify whether the meaning of work attributed by nurses in this research is present among other professionals. It is also recommended that institutions promote actions that contribute to the positive meaning of work.

## CONCLUSIONS

This study provided an understanding of the meaning of nurses’ work in a hospital context through value-based attributes that confer meaning, the most frequently reported being: affirmation; exchange of appreciation; unrestricted sharing of information; ability to trust; autonomy; altruistic reciprocity; interpersonal relationships; communication; conflict; interpersonal relationships with patients; reciprocity; engagement; happiness at work; opportunities for learning and development; recognition and moral integrity; affective commitment; local support; and time-related stress. In order to improve the performance of nurses in relation to the meaning of their work, a regular program of interaction with employees should be developed, providing the opportunity to address their individual needs.

Contributions to nursing and health care include knowledge of the meaning of work and recognition of intervening factors that permeate the feelings and emotions experienced at work and influence the meaning of work. In addition, it contributes to their development and self-fulfillment, helping them to achieve their life goals and purposes, thus fostering the formation of their professional and personal identity and improving nursing services, promoting the quality of care provided.
